# Chemotherapy use and survival in stage II nasopharyngeal carcinoma

**DOI:** 10.18632/oncotarget.21751

**Published:** 2017-10-11

**Authors:** Xin-Bin Pan, Shi-Ting Huang, Kai-Hua Chen, Xiao-Dong Zhu

**Affiliations:** ^1^ Department of Radiation Oncology, Cancer Hospital of Guangxi Medical University, Nanning, Guangxi 530021, P.R. China

**Keywords:** nasopharyngeal carcinoma, chemotherapy, survival, stage II

## Abstract

Although common, the use of chemotherapy for stage II nasopharyngeal carcinoma (NPC) is controversial due to its undefined clinical benefits. We therefore conducted a retrospective cohort study to investigate whether chemotherapy confers survival gains to stage II NPC patients. A total of 251 stage II (2010 UICC/AJCC staging system) NPC patients treated between January 2007 and December 2014 were retrospectively analyzed. Patients were matched using the propensity-score matching method. The primary endpoint was overall survival (OS). Secondary endpoints were locoregional-free survival (LRFS) and distant metastasis-free survival (DMFS). Our analyses revealed no significant differences in OS, LRFS, or DMFS for stage II NPC patients treated with radiotherapy (RT) alone, concurrent chemoradiotherapy (CCRT), or CCRT + adjuvant chemotherapy (AC). T2N1 (OR = 6.690; 95% CI, 3.091–14.481) and T1N1 (OR = 5.857; 95% CI, 2.278–15.061) patients were more likely to receive CCRT than T2N0 patients. Similarly, both T2N1 (OR = 10.513; 95% CI, 3.439–32.137) and T1N1 (OR = 7.321; 95% CI, 1.978–27.098) patients were more likely to receive CCRT + AC than T2N0 patients. The present matched survival analysis suggests potential overuse of chemotherapy in stage II NPC, as the addition of chemotherapy did not provide a survival benefit in this group of patients.

## INTRODUCTION

Nasopharyngeal carcinoma (NPC) is highly endemic in southern China [1, 2]. Concurrent chemoradiotherapy (CCRT) with or without adjuvant chemotherapy (AC) is superior to radiotherapy (RT) alone in the treatment of locoregionally advanced NPC [3–8]. Although CCRT with or without AC is recommended for stage II NPC by the National Comprehensive Cancer Network [9], several studies suggested that chemotherapy use does not improve survival compared with RT alone [10–17]. The Chinese Anti-Cancer Association recommends RT alone for the T2N0 subgroup. For the T1N1 and T2N1 subgroups, RT with or without chemotherapy is reasonable [11–14, 18, 19].

The incidence of stage II NPC has greatly increased with improvements in diagnosis. Although chemotherapy is not the standard treatment for stage II NPC, and its use remains controversial, a majority of stage II NPC patients are still receiving chemotherapy. Thus, it is possible that chemotherapy is overused in clinical practice without substantial survival gain. The objective of this retrospective cohort study was to assess whether chemotherapy use is associated with better survival in stage II NPC.

## RESULTS

A total of 251 stage II NPC patients were included in this study. Among them, 94 received RT alone, 103 received CCRT, and 54 received CCRT+AC. The last follow-up was in October 2016. Median follow-up time was 64 months (12–116 months). The follow-up rate was 96.81% with 8 patients lost.

### Chemotherapy and survival in stage II NPC

**Figure 1:**
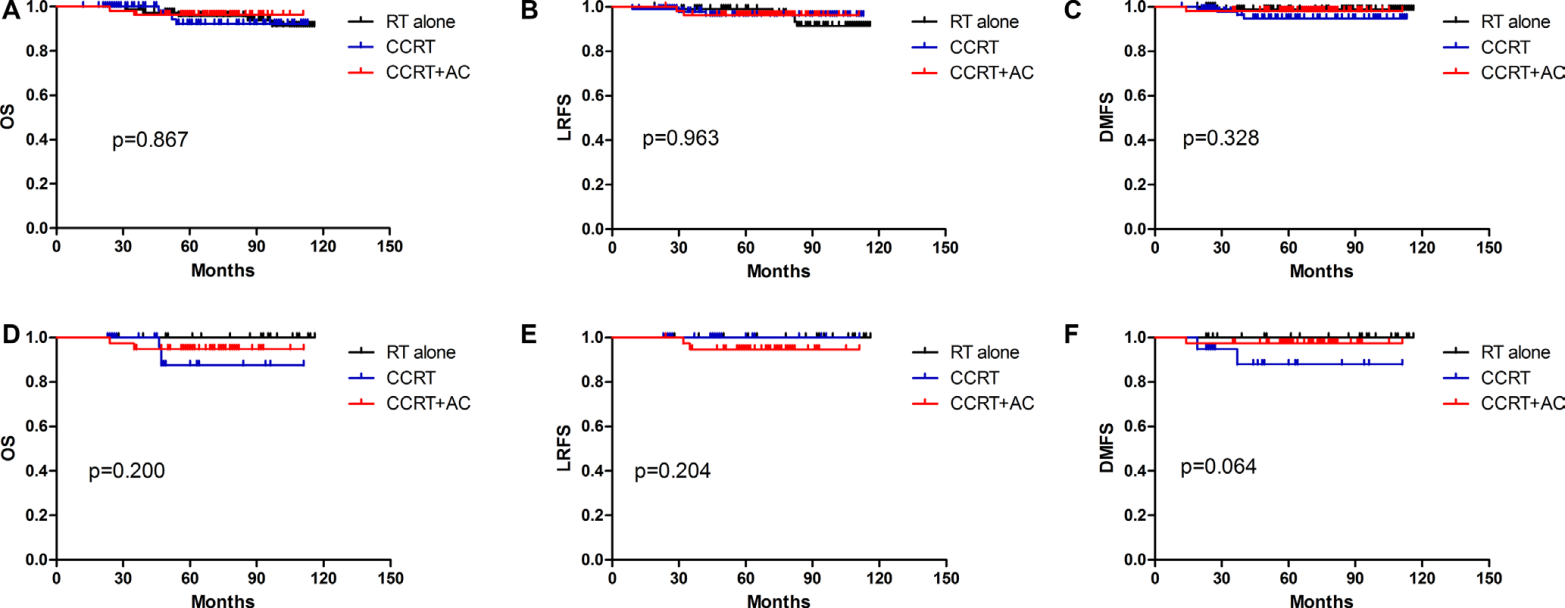
Kaplan–Meier survival curves of stage II nasopharyngeal carcinoma patients treated with radiotherapy (RT) alone, concurrent chemoradiotherapy (CCRT), and CCRT + adjuvant chemotherapy (CCRT+AC) in the unmatched cohort (**A**, **B**, **C**) and the propensity-matched cohort (**D**, **E**, **F**). Overall survival: OS; Locoregional-free survival: LRFS; Distant metastasis-free survival: DMFS.

The 5-year OS, LRFS, and DMFS of stage II NPC patients treated with RT alone, CCRT, and CCRT+AC are shown in Table 1. Survival curves are shown in Figure 1. Pairwise comparisons revealed no significant differences in treatment outcomes for patients receiving RT alone, CCRT, or CCRT+AC, in both the unmatched and the propensity-matched cohorts.

**Table 1:** Survival of stage II NPC patients treated with RT, CCRT, and CCRT+AC

	Unmatched cohort				Propensity-matched cohort			
	RT (*n* = 94)	CCRT (*n* = 103)	CCRT+AC (*n* = 54)	*P*	RT (*n* = 38)	CCRT (*n* = 38)	CCRT+AC (*n* = 38)	*P*
Age	44 (37.25, 55.75)	43 (38.00, 50.00)	43.5 (39.25, 48.00)	0.424	41 (36.25, 46.25)	41 (35.50, 50.50)	41.5 (36.75, 50.25)	0.773
Sex								
Female	30 (31.91%)	32 (31.07%)	17 (31.48%)	1.000	10 (26.32%)	13 (34.21%)	13 (34.21%)	0.722
Male	64 (68.09%)	71 (68.93%)	37 (68.52%)		28 (73.68%)	25 (65.79%)	25 (65.79%)	
Pathology								
WHO II	8 (8.51%)	9 (8.74%)	9 (16.67%)	0.261	5 (13.16%)	5 (13.16%)	5 (13.16%)	1.000
WHO III	86 (91.49%)	94 (91.26%)	45 (83.33%)		33 (86.84%)	33 (86.84%)	33 (86.84%)	
Technique								
IMRT	51 (54.26%)	87 (84.47%)	40 (74.07%)	0.001	28 (73.68%)	28 (73.68%)	28 (73.68%)	1.000
2D-CRT	43 (45.74%)	16 (15.53%)	14 (25.93%)		10 (26.32%)	10 (26.32%)	10 (26.32%)	
T stage								
T1	14 (14.89%)	22 (21.36%)	10 (18.52%)	0.513	10 (26.32%)	4 (10.53%)	4 (10.53%)	0.131
T2	80 (85.11%)	81 (78.64%)	44 (81.48%)		28 (73.68%)	34 (89.47%)	34 (89.47%)	
N stage								
N0	41 (43.62%)	11 (10.68%)	4 (7.41%)	0.001	4 (10.53%)	4 (10.53%)	4 (10.53%)	1.000
N1	53 (56.38%)	92 (89.32%)	50 (92.59%)		34 (89.47%)	34 (89.47%)	34 (89.47%)	
AJCC stage								
T1N1	14 (14.89%)	22 (21.36%)	10 (18.52%)	0.001	10 (26.32%)	4 (10.53%)	4 (10.53%)	0.342
T2N0	41 (43.62%)	11 (10.68%)	4 (7.41%)		4 (10.53%)	4 (10.53%)	4 (10.53%)	
T2N1	39 (41.49%)	70 (67.96%)	40 (74.07%)		24 (63.15%)	30 (78.94%)	30 (78.94%)	
Survival								
OS	95.9%	92.2%	96.3%	0.867	100.0%	87.5%	94.7%	0.200
LRFS	98.9%	96.1%	96.2%	0.963	100.0%	100.0%	94.6%	0.204
DMFS	98.7%	94.8%	98.1%	0.328	100.0%	88.0%	97.4%	0.064

Abbreviations - NPC: nasopharyngeal carcinoma, RT: radiotherapy, CCRT: concurrent chemoradiotherapy, AC: adjuvant chemotherapy, IMRT: intensity-modulated radiotherapy, 2D-CRT: two-dimensional conventional radiotherapy, OS: overall survival, LRFS: locoregional-free survival, DMFS: distant metastasis-free survival.

The 5-year OS, LRFS, and DMFS of T1N1, T2N0, and T2N1 patients treated with RT alone, CCRT, and CCRT+AC are shown in Table 2. Here again, CCRT and CCRT+AC did not improve survival compared to RT alone. The propensity score-matching method was not performed because of the limited sampling size of the three subgroups.

**Table 2:** Chemotherapy use and survival in T1N1, T2N0, and T2N1 subgroups

		RT	CCRT	CCRT+AC	*p*
T1N1	Total (***n*** **=** 46)	14 (30.43%)	22 (47.83%)	10 (21.74%)	
	OS	100.0%	100.0%	100.0%	1.000
	LRFS	100.0%	95.5%	100.0%	0.580
	DMFS	100.0%	100.0%	100.0%	1.000
T2N0	Total (***n*** **=** 56)	41 (73.22%)	11 (19.64%)	4 (7.14%)	
	OS	93.8%	100.0%	100.0%	0.654
	LRFS	97.5%	100.0%	100.0%	0.755
	DMFS	97.1%	90.0%	100.0%	0.556
T2N1	Total (***n*** **=** 149)	39 (26.17%)	70 (46.98%)	40 (26.85%)	
	OS	96.6%	88.4%	95.0%	0.873
	LRFS	100.0%	95.8%	94.9%	0.858
	DMFS	100.0%	94.4%	97.5%	0.383

Abbreviations - RT: radiotherapy, CCRT: concurrent chemoradiotherapy, AC: adjuvant chemotherapy, OS: overall survival, LRFS: locoregional-free survival, DMFS: distant metastasis-free survival.

### Survival among T1N1, T2N0, and T2N1 patients

The 5-year OS, LRFS, and DMFS for the T1N1, T2N0, and T2N1 subgroups are shown in Table 3. No significant differences were found after pairwise comparisons among the three subgroups within both the unmatched and the propensity-matched cohorts.

**Table 3:** Survival among T1N1, T2N0, and T2N1 subgroups

	Unmatched cohort				Propensity-matched cohort			
	T1N1 (*n* = 46)	T2N0 (*n* = 56)	T2N1 (*n* = 149)	*P*	T1N1 (*n* = 21)	T2N0 (*n* = 21)	T2N1 (*n* = 21)	*P*
Age	42 (38.00, 47.00)	45 (36.50, 54.25)	44 (39.00, 52.00)	0.516	42 (39.00, 47.00)	40 (35.00, 47.00)	41 (38.00, 47.00)	0.905
Sex								
female	12 (26.09%)	20 (35.71%)	47 (31.54%)	0.591	6 (28.57%)	6 (28.57%)	7 (33.33%)	1.000
male	34 (73.91%)	36 (64.29%)	102 (68.46%)		15 (71.43%)	15 (71.43%)	14 (66.67%)	
Pathology								
WHO II	4 (8.70%)	4 (7.14%)	18 (12.08%)	0.598	1 (4.76%)	0 (0.00%)	1 (4.76%)	1.000
WHO III	42 (91.30%)	52 (92.86%)	131 (87.92%)		20 (95.24%)	21 (100.00%)	20 (95.24%)	
Technique								
IMRT	30 (65.22%)	30 (53.57%)	118 (79.19%)	0.001	12 (57.14%)	11 (52.38%)	12 (57.14%)	1.000
2D-CRT	16 (34.78%)	26 (46.43%)	31 (20.81%)		9 (42.86%)	10 (47.62%)	9 (42.86%)	
Treatment								
RT	14 (30.43%)	41 (73.22%)	39 (26.17%)	0.000	9 (42.86%)	9 (42.86)	9 (42.86%)	0.966
CCRT	22 (47.83%)	11 (19.64%)	70 (46.98%)		9 (42.86%)	8 (38.09%)	10 (47.62%)	
CCRT+AC	10 (21.74%)	4 (7.14%)	40 (26.85%)		3 (14.28%)	4 (19.05%)	2 (9.52%)	
Survival								
OS	100.0%	95.6%	93.5%	0.165	100.0%	94.4%	100.0%	0.509
LRFS	97.8%	98.2%	96.6%	0.588	95.2%	95.2%	100.0%	0.999
DMFS	100.0%	95.9%	96.9%	0.500	100.0%	94.7%	100.0%	0.378

Abbreviations - RT: radiotherapy, CCRT: concurrent chemoradiotherapy, AC: adjuvant chemotherapy, IMRT: intensity-modulated radiotherapy, 2D-CRT: two-dimensional conventional radiotherapy, OS: overall survival, LRFS: locoregional-free survival, DMFS: distant metastasis-free survival.

### Chemotherapy use in stage II NPC

For stage II NPC, 41.0% of the patients received CCRT, 21.5% received CCRT+AC, and 37.5% received RT alone. The percentage of patients receiving RT alone, CCRT, and CCRT+AC were, respectively, 30.43%, 47.83%, and 21.74% for T1N1; 73.22%, 19.64%, and 7.14% for T2N0; and 26.17%, 46.98%, and 26.85% for T2N1 (Table 3).

With the T2N0 subgroup as reference, T1N1 patients were more likely to receive CCRT (OR = 5.857; 95% CI, 2.278–15.061) and CCRT+AC (OR = 7.321; 95% CI, 1.978–27.098). Similarly, for T2N1 patients, the likelihood of receiving CCRT (OR = 6.690; 95% CI, 3.091–14.481) and CCRT+AC (OR = 10.513; 95% CI, 3.439–32.137) was higher than for T2N0 patients (Table 4).

**Table 4:** Likelihood of receiving chemotherapy in the T1N1, T2N0, and T2N1 subgroups

	CCRT		*p*	OR (95% CI)	CCRT+AC		*P*	OR (95% CI)
	yes	No			yes	no		
T2N0	11	41		1 [Reference]	4	41		1 [Reference]
T1N1	22	14	0.000	5.857 (2.278–15.061)	10	14	0.003	7.321 (1.978–27.098)
T2N1	70	39	0.000	6.690 (3.091–14.481)	40	39	0.000	10.513 (3.439–32.137)

Abbreviations - CCRT: concurrent chemoradiotherapy, AC: adjuvant chemotherapy, OR: odds ratio, CI: confidence interval.

## DISCUSSION

Our study indicated no significant differences in OS, LRFS, and DMFS between stage II NPC patients treated with RT alone, CCRT, and CCRT + AC. Accordingly, our results suggest overuse of CCRT and AC for stage II NPC treatment.

Chemotherapy is not a standard treatment option for stage II NPC, and its use in these patients remains controversial. It was reported that survival of stage II NPC patients treated with 2D-CRT alone was not satisfactory [20–22]. In contrast, others suggested that chemotherapy addition translates into substantial improvements in DMFS and long-term OS [9, 23, 24]. However, these investigations used various TNM staging systems (Chinese 1992 or AJCC 2002) and T3 or N2 (AJCC 2010) patients might have been included. Thus, the referred results should be treated with caution. IMRT was expected to improve survival, but superiority of IMRT over 2D-CRT was not conclusively proved [25–29]. In fact, compared to 2D-CRT, IMRT only improved the quality of life in stage II NPC patients [30]. Luo et al. reported that CCRT had higher 3-year OS than IMRT alone (100.0% vs 81.4%, *P* = 0.04) [31]. Moreover, a multi-center study suggested that CCRT improved 5-year LRFS and progression-free survival [32]. However, several retrospective cohort studies showed that IMRT alone was a reasonable option for stage II NPC whereas, in line with the present results, chemotherapy use failed to improve survival [10–12, 15–17].

Stage II NPC is divided into three subgroups (T1N1, T2N0, and T2N1). Our subgroup analysis results showed that compared to RT alone, CCRT and CCRT+AC did not improve survival in T1N1, T2N0, or T2N1. However, Guo et al. reported that chemotherapy improved LRFS in T1N1 [11]. In that report, the LRFS of T1N1 patients receiving chemotherapy or RT alone was 97.0% and 91.3% (*p* = 0.017), respectively, although the improvement in LRFS did not translate into better DMFS or OS. Considering that no DMFS and OS benefits were achieved by chemotherapy, it may be appropriate to remove chemotherapy from T1N1 cases. In case of locoregional recurrence after RT alone, salvage treatments were still effective to control disease [33, 34].

The prognosis of T1N1, T2N0, and T2N1 cases remains controversial. Xiao et al. reported that the 5-year OS for T1N1, T2N0, and T2N1 patients was 91.3%, 85.8%, and 73.1% (*p* < 0.05) respectively [21]. Luo et al. also indicated that 3-year OS was significantly poorer in T2N1 than in T1N1 and T2N0 patients (74.5 vs 100.0%; *P* = 0.01) [31]. Based on these data, T2N1 patients appear to constitute a unique subgroup characterized by worse survival. Potential interpretations of this phenomenon include: (1) Parapharyngeal extension increases the risk of distant metastasis [35–37], as 5-year DMFS of stage II NPC patients with parapharyngeal extension was reported to be 12.6% lower than in patients without this complication (73.6% vs. 86.2%) [37]. (2) N1 tumors carry a high risk of distant metastasis. Tang et al. reported that DMFS would decrease further when parapharyngeal extension occurred concurrently with positive lymph node metastasis (T2N1) [38]. According to these interpretations, clinicians might administrate more chemotherapy to T2N1 patients.

However, our study showed that 5-year DMFS for T1N1, T2N0, and T2N1 patients was 100.0%, 95.9%, and 96.9% (*p* = 0.500). Moreover, no differences were found between subgroups in 5-year LRFS and OS. Of note, several studies showed similar results [10, 11, 14, 15]. Yet, despite much evidence that chemotherapy did not improve survival of stage II NPC, it was still widely used in clinical practices. Indeed, our data showed that the likelihood of receiving chemotherapy was higher for both T2N1 and T1N1 than for T2N0.

Our findings have significant clinical and economic implications. First, patients who received CCRT or CCRT+AC showed more acute and late toxicity reactions without a substantial survival benefit [8–10]. Second, compared to RT alone, CCRT impaired the quality of life of stage II NPC patients [39]. Third, because the cost of chemotherapy for NPC is high, overuse of chemotherapy imposes a considerable economic burden on society, especially in developing countries such as China [39].

Our study has some limitations that are worth considering: (1) Although we used the propensity score-matching method to reduce biases caused by confounding variables, confounding factors may still have influenced this retrospective cohort study. (2) The risk of treatment failure was very low in stage II NPC in the first five years. Thus, the follow-up time of this study might be insufficient for properly assessing survival. We are going to conduct a prospective, randomized control clinical trial (NCT02116231) to compare IMRT alone with IMRT and concurrent chemotherapy for the treatment of stage II NPC. The results of this trial should clarify whether addition of chemotherapy provides further survival benefits in this subset of patients.

In conclusion, our study highlights a potential overuse of chemotherapy in the treatment of stage II NPC, as its addition to radiotherapeutic regimes did not result in survival improvement.

## MATERIALS AND METHODS

### Study population

A retrospective analysis was conducted in untreated NPC patients examined at the Cancer Hospital of Guangxi Medical University from January 2007 to December 2014. Patients without complete pretreatment evaluations, including pathology, nasopharyngoscopy, magnetic resonance imaging (MRI) or computed tomography (CT) scan of the nasopharynx and neck, chest radiography or CT scan, abdominal sonography or CT scan, and whole-body bone scan were excluded. Patients were restaged according to the 2010 International Union Against Cancer/American Joint Committee on Cancer (UICC/AJCC) staging system [40].

### Radiotherapy and chemotherapy

A detailed description of the radiotherapy modalities was published recently [29]. Total doses for gross tumor volumes were 66 Gy to 70 Gy for two-dimensional conventional radiotherapy (2D-CRT). The prescribed radiation doses of intensity-modulated radiotherapy (IMRT) were 66 Gy to 70.06 Gy for gross tumor volumes, and 54 Gy to 60 Gy for clinical tumor volumes.

Concurrent chemotherapy was scheduled on days 1, 22, and 43 with 80 to 100 mg/m^2^ of cisplatin for 1 or 3 days per cycle during radiotherapy. AC was 80 to 100 mg/m^2^ of cisplatin for 1 or 3 days, and 600–750 mg/m^2^/d of 5-fluorouracil in continuous intravenous infusion for 96 hours or 120 hours in a cycle of 28 days for 2 to 3 cycles. Chemotherapy was postponed or discontinued in patients who experienced serious toxicity and could not recover before the next scheduled cycle.

### Endpoints and follow-up

The primary endpoint was overall survival (OS). Secondary endpoints were locoregional-free survival (LRFS) and distant metastasis-free survival (DMFS). OS, LRFS, and DMFS were defined as the time interval from the first day of treatment until, respectively, the time of death, nasopharyngeal or regional lymph node relapse, or distant metastasis.

Patients were followed up every 3 months through the first 2 years, every 6 months for the next 3 years, and then annually. Physical examination, nasopharyngoscopy with/without biopsy, MRI or CT scan of the nasopharynx and neck, chest radiography or CT scan, and abdominal sonography or CT were performed. Bone scan was conducted if clinically indicated.

### Statistical analysis

Continuous data were analyzed by student's *t*-test. Categorical variables were analyzed by chi-squared or Fisher's exact test. Survival was assessed using Kaplan–Meier plots with log-rank test statistics.

Based on the propensity score matching method, one-to-one nearest-neighbor matching was adopted to overcome selection bias among groups by use of a 0.1 caliper. The propensity score calculated by a logistic regression model represents the probability of each patient being assigned to each treatment group. Variables likely influencing survival, including age, sex, pathology, RT technique, T-stage, N-stage, clinical stage, and treatment modality, were used in the score-matching method.

Statistical analyses were performed by IBM SPSS Statistics Version 23.0 (IBM Co., Armonk, NY, USA). Two-tailed *P* < 0.05 were considered statistically significant.

### Ethical statement

This study was approved by the Ethics Committee of the Cancer Hospital of Guangxi Medical University. Informed consent was obtained from the patients and/or guardians.

## Abbreviations

NPC: nasopharyngeal carcinoma
RT: radiotherapy
CCRT: concurrent chemoradiotherapy
AC: adjuvant chemotherapy
IMRT: intensity-modulated radiotherapy
2D-CRT: two-dimensional conventional radiotherapy
OS: overall survival
LRFS: locoregional-free survival
DMFS: distant metastasis-free survival
